# Arterial Stiffness as a Surrogate Marker of Cardiovascular Disease and Atherosclerosis in Patients with Arthritides and Connective Tissue Diseases: A Literature Review

**DOI:** 10.3390/diagnostics13111870

**Published:** 2023-05-26

**Authors:** Konstantinos Triantafyllias, Leif-Erik Thiele, Lorenzo Cavagna, Xenofon Baraliakos, George Bertsias, Andreas Schwarting

**Affiliations:** 1 Rheumatology Center Rhineland-Palatinate, Kaiser-Wilhelm-Str. 9-11, 55543 Bad Kreuznach, Germany; 2 Department of Internal Medicine I, Division of Rheumatology and Clinical Immunology, University Medical Center, Johannes Gutenberg University, 55131 Mainz, Germany; 3 Department of Rheumatology, University and IRCCS Policlinico S. Matteo Foundation Pavia, 27100 Pavia, Italy; 4 Rheumazentrum Ruhrgebiet Herne, Ruhr-University Bochum, 44649 Herne, Germany; 5 Department of Internal Medicine and Rheumatology, School of Medicine, University of Crete, 71500 Heraklion, Greece

**Keywords:** cardiovascular disease, arterial stiffness, atherosclerosis, pulse wave velocity, augmentation index, rheumatoid arthritis, psoriatic arthritis, systemic lupus erythematosus, systemic sclerosis

## Abstract

The increased cardiovascular (CV) risk among patients with autoimmune rheumatic diseases, such as arthritides and connective tissue diseases, has been extensively documented. From a pathophysiological standpoint, systemic inflammation in the context of the disease can lead to endothelial dysfunction, accelerated atherosclerosis, and structural changes in vessel walls, which, in turn, are associated with exaggerated CV morbidity and mortality. In addition to these abnormalities, the increased prevalence of traditional CV risk factors, such as obesity, dyslipidemia, arterial hypertension, and impaired glucose metabolism, can further worsen the status of and overall prognosis for CV in rheumatic patients. However, data on appropriate CV screening methods for patients with systemic autoimmune diseases are scarce, and traditional algorithms may lead to an underestimation of the true CV risk. The reason for this is that these calculations were developed for the general population and thus do not take into account the effect of the inflammatory burden, as well as other chronic-disease-associated CV risk factors. In recent years, different research groups, including ours, have examined the value of different CV surrogate markers, including carotid sonography, carotid–femoral pulse wave velocity, and flow-mediated arterial dilation, in the assessment of CV risk in healthy and rheumatic populations. In particular, arterial stiffness has been thoroughly examined in a number of studies, showing high diagnostic and predictive value for the occurrence of CV events. To this end, the present narrative review showcases a series of studies examining aortic and peripheral arterial stiffness as surrogates of all-cause CV disease and atherosclerosis in patients with rheumatoid and psoriatic arthritis, as well as in systemic lupus erythematosus and systemic sclerosis. Moreover, we discuss the associations of arterial stiffness with clinical, laboratory, and disease-specific parameters.

## 1. Introduction

The majority of rheumatic diseases are characterized by chronic and systemic inflammatory processes with autoimmune origins. Although the exact pathophysiological mechanisms associated with these diseases are not fully understood, it is assumed that interactions between predisposing genetic factors and triggering environmental parameters can lead to a dysregulation of the immune system and generalized tissue damage, in addition to the effect of various autoantibodies and other soluble mediators [1].

Systemic inflammatory diseases, such as arthritides, connective tissue diseases, and vasculitides, have been found to be associated with increased cardiovascular (CV) risk, as well as with higher CV-associated morbidity and mortality rates [2,3,4,5,6,7]. In particular, for prototype rheumatic conditions such as rheumatoid arthritis (RA), the magnitude of this excess risk appears to be comparable to that reported for patients with diabetes mellitus [8]. Moreover, the risk of CV mortality in patients with systemic lupus erythematosus (SLE) is elevated 1.5- to nearly 3-fold compared to the general population [9,10,11]. Accumulating evidence has also shown increased CV morbidity and mortality in further rheumatic conditions, such as systemic sclerosis, Sjögren’s syndrome, and several arthritides (i.e., gout, psoriatic arthritis (PsA)) and vasculitides [12,13]. In this regard, chronic inflammation is a well-recognized modulator of endothelial function and arterial stiffness, leading to accelerated atherosclerosis and subsequently higher rates of CV events [14].

In the general population, CV risk can be calculated via traditional assessment scores (the Framingham risk score, PROCAM, Systematic Coronary Risk Evaluation (SCORE)) based on CV risk factors, such as hyperlipidemia, arterial hypertension, body mass index (BMI), and nicotine consumption [15,16,17]. However, these scores tend to underestimate the CV risk in the case of systemic rheumatic diseases, given that disease-associated aspects, such as the cumulative inflammatory burden, the effects of glucocorticoids (and other medications), and patients’ reduced physical fitness, are not taken into account. According to the European League Against Rheumatism (EULAR), CV assessment of patients with inflammatory joint diseases is suggested at least once every five years and after major changes in anti-inflammatory therapy [13]. Other recommendations include optimizing disease activity control, lifestyle improvements, and screening for asymptomatic atherosclerotic plaques by use of carotid ultrasound and a 1.5-multiplied SCORE [13,18]. However, additional risk calculators that are recommended for use in RA patients (the 1.5 SCORE multiplier, the Expanded Cardiovascular Risk Prediction Score for RA, and QRISK2) did not predict CV risk more accurately than CV disease (CVD) risk calculators developed for the general population [19]. Similarly, CVD risk tools that consider a diagnosis of SLE showed contradictory results regarding the predictive accuracy for CVD development when compared to the existing traditional tools [20,21]. For this reason, there is a need for additional biomarkers that can help us achieve a more accurate assessment of true CV risk [22].

**Figure 1:**
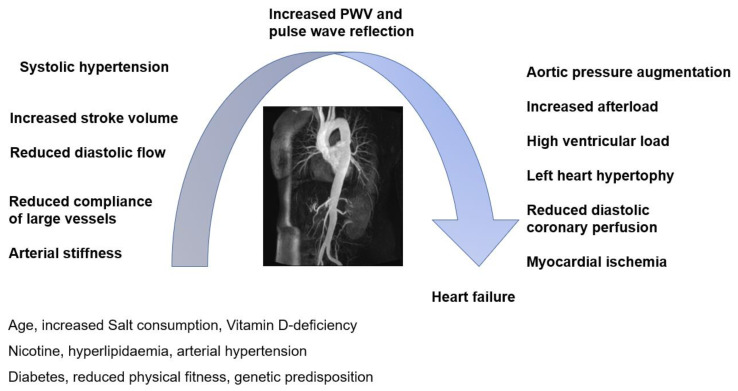
Cardiovascular interplay (adapted from [23]).

Stiffness of the large arteries is one of the best-validated CV surrogates in the general population, showing a high positive predictive value for future CV events, next to traditional CV risk factors (Figure 1) [14,23]. Even though a significant number of studies examine arterial stiffness in cohorts of patients with rheumatic diseases, longitudinal data regarding its diagnostic accuracy are scarce.

Arterial stiffness is modifiable and can independently predict CV risk in the general population. It can be measured using several methods, such as oscillometry (pulse wave velocity (PWV); gold standard), echocardiography (i.e., the aortic stiffness index (AoSI), aortic distensibility, pulse wave analysis (i.e., the augmentation index 75 (AIx@75)), and pressure–strain elastic modulus (PSEM) (Table 1) [24,25,26,27,28,29].

**Figure 2:**
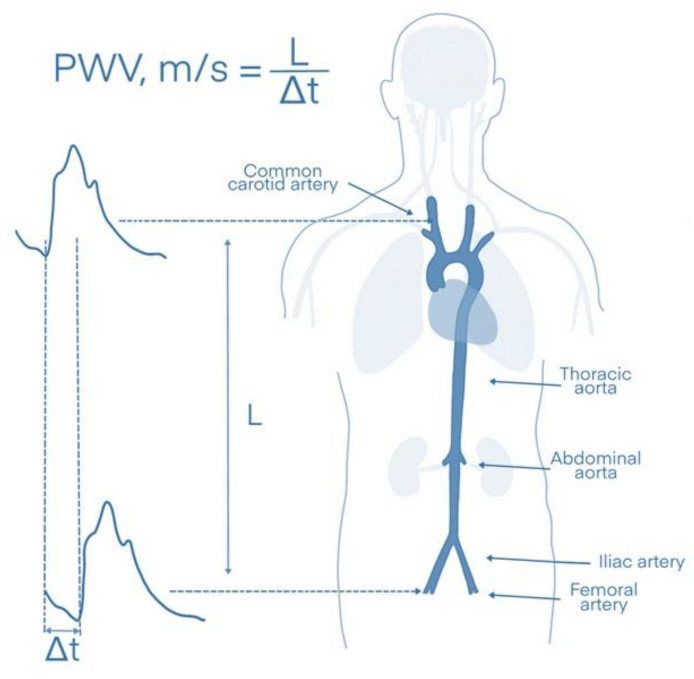
Measurement of aortic stiffness via the carotid–femoral pulse wave velocity (cfPWV). L: distance between the carotid and femoral arteries, Δt: time needed for the pulse wave to travel between the two measurement points.

In contrast to parameters such as blood pressure, lipids, or glucose levels, which correspond to the instantaneous intensity of CV risk factors (and can thus fluctuate significantly over time), arterial stiffness reflects the long-term effects of established and unknown risk factors, as well as the individual genetic predisposition of each person [22]. In particular, stiffness of the aorta, a modifiable marker that can be measured via the carotid–femoral PWV (cfPWV), has been shown to have a high predictive value for CV events in the general population; it is considered the gold standard for the measurement of aortic stiffness (Figure 2) [30].

In recent years, our research group and others have examined aortic stiffness, as measured by cfPWV, in the context of various systemic rheumatic diseases, including RA [31], mixed connective tissue disease [32], SLE [33], antisynthetase syndrome [34], and the chronic pain syndrome fibromyalgia [35]. Since data on the surrogate markers of CV risk in patients with rheumatic diseases are limited, the aim of this review is to provide information from studies examining arterial stiffness as a marker of all-cause and atherosclerotic disease in patients with systemic inflammatory rheumatic conditions. We thus present data on both peripheral and aortic stiffness. Moreover, we aim to collect and present the published data on the associations of arterial stiffness with clinical, laboratory, and disease-specific parameters.

In the present work (part 1), we discuss prototype arthritides, such as RA and PsA, as well as connective tissue diseases with increased CV risk and high CV mortality rates, such as SLE and SSc. In the second part of the review, we focus on data regarding arterial stiffness in patients with further rheumatic diseases, such as systemic vasculitides.

## 2. Methods

For this narrative review, a search strategy was developed to enable us to identify the most important literature on the topic. An exhaustive search was performed on PubMed/Medline and Google Scholar using a keyword combination according to the Boolean operators: “arterial stiffness” AND (rheumatoid arthritis OR psoriatic arthritis OR systemic lupus erythematosus OR systemic sclerosis) (1044); as well as “aortic stiffness” AND (rheumatoid arthritis OR psoriatic arthritis OR systemic lupus erythematosus OR systemic sclerosis) (734); as well as (“pulse wave velocity”) AND (rheumatoid arthritis OR psoriatic arthritis OR systemic lupus erythematosus OR systemic sclerosis) (1346). This produced a total of 3124 publications. Limiting the results to publications from the past two decades left us with 3101 articles across all keyword combinations. We then manually excluded irrelevant and duplicated papers, as well as papers without full text, by skimming over the titles and, where necessary, the abstracts. Moreover, we ensured that this methodology was in accordance with that described by Gasparyan et al. (narrative review) [36].

## 3. Arterial Stiffness

### 3.1. Definition of Arterial Stiffness

Arterial stiffness refers to the loss of arterial compliance. It is a major independent risk factor for CV complications leading to systolic hypertension and increased pulse pressure in the microvasculature of target organs [37]. Moreover, it is an important determinant of vascular aging and independently predicts CV events, such as myocardial infarction, atherosclerosis, and stroke [27,38].

### 3.2. Pathogenesis of Arterial Stiffness

The process of arterial stiffening is characterized by alterations in the extracellular matrix composition involving the fragmentation of elastin fibers, the deposition of stiff wall materials, such as collagen fibers, and the cross-linking of collagen molecules by advanced glycation end-products (AGEs) [39]. Such pathophysiological processes can be caused by aging and arterial hypertension [39]. In the latter case, intraluminal forces such as pulse pressure lead to a stretching of the arterial wall, causing an increase in arterial stiffness [39,40]. In addition, the activation of the renin–angiotensin system leads to structural alterations in the arterial walls via vascular smooth-cell proliferation, mild inflammation, collagen increases, and the formation of AGEs [40].

However, in addition to structural stiffening, functional vascular stiffening caused by endothelial dysfunction also plays an important role. NO synthesis and endothelial NOS activity (and content) can be decreased by pathophysiologic mechanisms that are associated with wall architecture changes and thus further promote arterial stiffening [39]. Even if atherosclerosis is thought to be linked to arterial stiffness, this link has not been clearly established, mainly due to their complex interplay [39]. In pathophysiological terms, arterial stiffening is distinct from atherosclerosis since atherosclerosis typically involves the intima layer and is characterized by lipid accumulation, the migration of smooth muscle cells and inflammatory cells, and foam cell development [39]. On the other hand, both atherosclerosis and arterial stiffness often coexist in the same parts of the vascular tree and share some common pathogenetic mechanisms, such as insulin resistance and impaired endothelial function [39].

### 3.3. Methods of Assessment of Aortic/Arterial Stiffness

Arterial stiffness can be assessed using a variety of methods (Table 1). The measurement methodologies of these markers are described below:

- Pulse wave velocity (PWV): The most widely used measure of arterial stiffness is an oscillometric marker called PWV. PWV reflects the speed of arterial pulse waves traveling along the aorta and other parts of the arterial tree and is usually calculated by dividing distance by the pressure wave transit time at the two points of the measured arteries [41,42]. For example, aortic PWV (or cfWV) is measured between the carotid and femoral arteries in meters (the distance between the carotid and femoral arteries) per second (the time one pulse wave needs to cover this distance; Δs/Δt) [31].
- The augmentation index (AIx): Another technique uses applanation tonometry, which is widely used to evaluate the central pressure and wave reflections through the non-invasive use of a marker called AIx [43]. AIx, measured as a %, represents the supplementary increase in systolic blood pressure that is mainly caused by wave reflections. It is calculated as the ratio between (1) the difference between peak systolic pressure and the shoulder of the ascending part of the blood pressure curve, and (2) the pulse pressure [43].
- The aortic stiffness index (AoSI): This parameter can be measured by Doppler echocardiography at the level of the aortic root by assessing systolic (AoS) and diastolic (AoD) aortic diameters 3 cm above the aortic valve. It uses the following formula: AoSI = ln(SBP/DBP)/(AoS − AoD/AoD) (where SBP = systolic blood pressure and DBP = diastolic blood pressure) [44,45].
- The stiffness parameter beta, or β-stiffness index, is a local elastic parameter that is usually assessed by carotid ultrasound in the longitudinal plane coupled with arterial pressure data derived from applanation tonometry. It is calculated using the following formula: β-stiffness index  =  ln(SBP/DBP)/[(Ds − Dd)/Dd] (where Ds = systolic diameter; Dd = diastolic diameter) [46]. Thus, the β-stiffness index and AoSI are similar markers describing the stiffness of different arteries.
- The pressure–strain elastic modulus (PSEM): PSEM is a variation of AoSI and is calculated using the same formula as AoSI. However, it includes pressure values converted from mmHg to Pascal units [47].
- Aortic distensibility is another echocardiographic measure that is used to describe arterial stiffness. It is calculated using the following formula: aortic distensibility = (2×)/(AoS − AoD)/AoD/(SBP − DBP) [45].
- The cardio-ankle vascular index (CAVI) was developed to further improve the PWV method by making it less dependent on arterial pressure. To this end, the brachial–ankle PWV is measured and inserted into the following equation: CAVI = a[(2ρ/(SBP − DBP)) × ln(SBP/DBP) × PWV^2^] + b (a and b are constants; ρ= blood density) [48].

### 3.4. Diagnostic Accuracy in the General Population

According to the position paper from the European Society of Cardiology’s working group on peripheral circulation, which was endorsed by the Association for Research into Arterial Structure and Physiology (ARTERY) Society, measurements of carotid–femoral (aortic) stiffness using cfPWV and of brachial–ankle stiffness using baPWV can be useful for primary and secondary CVD prevention in the general population (recommendation and level of evidence: IIa/A and IIb/B, respectively) [49]. Regarding cfPWV, two studies and an independent meta-analysis revealed that patients at intermediate risk could be reclassified into a higher or lower CV risk category when cfPWV was measured [50,51,52]. cfPWV showed additional predictive value for the risk of CVD above traditional CV risk factors, known CV risk scores (i.e., SCORE), and pulse pressure or other measures of atherosclerosis [50,51,52]. In a further meta-analysis (*n* = 17,635 participants), the 5-year overall net reclassification index for coronary heart disease and stroke in intermediate risk subjects was 14.8% and 19.2%, respectively [27]. 

Regarding baPWV, a meta-analysis including 8,169 participants showed that an increase in the baPWV of 1 m/s was associated with increases of 12%, 13%, and 6% in CV events, CV mortality, and all-cause mortality, respectively [53]. Thus, the predictive value of this marker has been described. 

PWV measurements of other arterial segments (regional or local) as other markers of aortic stiffness, such as CAVI or echocardiographic indices, are presented in the same guidelines as additional promising biomarkers [49,54]. However, these have not been validated to the same extent as cfPWV. For example, CAVI has the theoretical advantage of being less dependent on BP levels and has been correlated with several arteriosclerotic and atherosclerotic diseases [49,55]. Moreover, echocardiographic indices can be assessed in multiple clinical settings. 

## 4. Utility of Aortic/Arterial Stiffness in Predicting the CV Risk in Rheumatic Disease Patients

### 4.1. Direct Evidence

Prospective data on the diagnostic accuracy of arterial stiffness in patients with rheumatic diseases are scarce. The vast majority of the existing studies examine the associations of arterial stiffness at one time point during the course of the disease, and thorough longitudinal data examining the predictive value of this marker are lacking. In the setting of RA, we were able to identify only two prospective studies that related arterial stiffness measures to future outcomes [56,57] (Table 2). In the study of Cioffi et al., 226 RA patients were examined by AoSI via echocardiography and were found to have greater stiffness of the ascending aorta compared with 226 matched controls [57]. Multivariate Cox regression analysis revealed that abnormally high AoSI values independently predicted death or all-cause hospitalization by a hazard ratio of 2.85 (95% CI 1.03–7.85) during a 12-month follow-up period. In the study conducted by Ikdahl et al., 138 RA patients underwent measurements of vascular biomarkers (aortic PWV, carotid intima-media thickness (cIMT), and AIx) in 2007, and were subjected to clinical and laboratory examinations [56]. The occurrence of CVD events was recorded six years later (2013). In crude Cox PH regression analyses, aPWV (*p* < 0.001) and cIMT (*p* < 0.001) were predictive of CVD events, as were age (*p* = 0.01), statin (*p* = 0.01), and glucocorticoid use (*p* = 0.01). However, AIx was nonsignificant (*p* = 0.19). Two further prospective studies evaluated the predictive value of arterial stiffness for the occurrence of digital ulcerations and disease progression in patients with SSc and are included in Table 2 [58,59].

### 4.2. Indirect Evidence

#### 4.2.1. Rheumatoid Arthritis

RA is a common chronic inflammatory disease, which affects mostly individuals during late adulthood [60]. This disease manifests in chronic symmetric polyarthritis, morning stiffness, and, in some cases, the involvement of internal organs such as the lungs [60]. Laboratory examinations show increased inflammatory activity and the presence of specific antibodies, such as rheumatoid factor (RF) and antibodies against cyclic citrullinated peptide (anti-CCP). The complications of RA include joint deformities, especially in the fingers, wrists, and feet, but also in larger joints. Moreover, individuals with RA have been shown to be associated with a 1.5–2-fold higher CV risk than age- and sex-matched individuals from the general population [60,61].

Patients with chronic RA disease courses are more likely to have stiffer arteries compared to healthy controls, as indicated by studies on PWV [62,63,64,65,66,67,68]. Data on other surrogate markers, such as elevated augmentation pressure [69] and low aortic distensibility [67], are also indicative of higher levels of arterial stiffness in patients with RA compared with the general population.

One of the main contributors to arterial stiffness in RA patients and healthy subjects is increased age, which has been shown to be significantly associated with higher levels of arterial stiffness in multiple RA cohorts, as well as in subjects without autoimmune diseases [31,65,66,70,71,72]. In pathophysiological terms, this can be explained by a change in the properties of the aortic wall during the course of a lifetime: aging is followed by a gradual replacement of the elastic component elastin with inelastic collagen fibers [73].

Furthermore, several RA disease activity and chronicity parameters have been shown to be associated with higher levels of arterial stiffness, even though data on these associations are partially contradictory [62,66,69,72,74]. PWV has been found to correlate with C-reactive protein (CRP) (*p* = 0.03 after adjustment for age in a study of 90 RA patients) and the erythrocyte sedimentation rate (ESR) [66], as well as with disease duration [72,74] in several studies. However, data from further case-control studies show the opposite [62,74]. Similarly, in the work of Botta et al., when the clinical score DAS28 was used as a disease activity parameter (with a cut-off value of 3.2), significantly higher PWV values were found in active vs. inactive RA patients [66]. Two further explorations were nevertheless unable to show significant associations between DAS28 and any arterial stiffness parameters [67,71]. Interestingly, in one of the studies conducted by our research group, the number of tender—rather than swollen—joints was found to be associated with a higher cfPWV, indicating the possible role of pain in the development of arterial stiffness/stiffening in patients with RA (0.051 95% CI (0.008–0.207), p_adj_ = 0.034) [31]. Taverner et al. also identified an association between tender joints and another marker of arterial stiffness (AIx; β = −0.33; *p* = 0.045) in a cohort of 214 patients, supporting a possible pathophysiological association between vascular stiffening and pain [71]. In the work of Gunter et al., different vascular parameters (including PWV, AIx, and pressure pulsatility) were found to be associated with both traditional CV risk factors and RA characteristics in 177 RA patients without established CVD [75].

Byram et al. examined the effect of exercise on arterial stiffness in patients with RA [76]. Briefly, RA patients who exercised regularly (patient-reported exercise; median 113 min/week (IQR: 60, 210)) were compared with their non-exercising RA counterparts using a cross-sectional study design. Age, ethnicity, gender, and disease duration did not significantly differ between the two groups (all *p* > 0.05). Exercise was found to be associated with lower CRP, ESR, DAS28, and PWV values, but also with a lower prevalence of hypertension. Moreover, a positive association with higher-density lipoprotein (HDL) was observed [76]. However, the overall standard lipid profile and body mass index (BMI) were not significantly different between the two groups. The authors stated that exercise could play a cardioprotective role in the setting of RA due to its association with a decreased prevalence of traditional CV risk factors [76].

Another important area of research in RA focuses on the effects of patient medication on arterial stiffness. Some of the studies performed on this topic examine the effects of immunosuppressants or biological agents on patients’ vascular status, while others focus on traditional antihypertensive drugs. For instance, regular therapy regimes for RA using tumor necrosis factor inhibitors (TNF inhibitors) were reviewed with respect to their effect on arterial stiffness. In a small cohort, Maki-Petaja et al. showed a lowering effect on aortic inflammation and stiffness (cfPWV: from 9.09 ± 1.77 to 8.63 ± 1.42 m/s, *p* = 0.04), but not on AIx or aortic pressure, after 8 weeks of treatment with either Adalimumab or Etanercept [77]. Other studies reported no lowering of arterial stiffness surrogates from this kind of medication but did report lower stiffness progression [44,78] when compared to therapy with other DMARDs [44].

The effects of other immunosuppressants, such as Rituximab (a B-cell-depleting agent), have also been examined in this regard. Mathieu et al. did not find an improvement in arterial stiffness (PWV, AIx) after 6 and 12 months of therapy with Rituximab in 33 patients with RA (PWV: 8.1 (3.1) m/s at baseline, 8.1 (2.8) at 6 months, 8.0 (2.7) at 1 year, *p* = 0.924 and AIx: 30.4 (8.2)% at baseline, 28.6 (7.6) at 6 months, 29.4 (6.7) at 1 year, *p* = 0.216) [79]. Similarly, in the study of Novikova et al., no improvement in arterial stiffness and arterial resistance indices after 24 weeks of Rituximab treatment was shown in 55 female patients with RA [80]. Interestingly, treatment with glucocorticoids (GC) has been shown to be independently associated with increased arterial stiffness in the context of RA [63]. GC can cause an increase in serum glucose, serum lipids, and blood pressure, and it could thus increase CV risk. However, these effects are mainly observed in cases of high-dose GC [81].

Regarding the effect of antihypertensive drugs on arterial stiffness, Enalapril was found to have no lowering effect on cfPWV in a study of 27 patients with RA who were compared with 26 controls receiving a placebo for 12 weeks [82]. However, this medication was shown to have a significant lowering effect on another marker of arterial stiffness (CAVI), which is calculated using the heart–ankle PWV from the origin of the aortic valve to the ankle region in combination with blood pressure [82,83]. A reduction in the CAVI from 7.4 ± 1.2 to 7.1 ± 0.9 (delta CAVI: 0.21) was found in the Enalapril group, whereas a delta increase of 0.39 was observed in the placebo group.

Follow-up examinations of arterial stiffness are important, as increases in arterial stiffness during a patient’s lifetime are associated with worse CV outcomes [27]. Ikdahl et al. showed significant correlations of both cfPWV (*p* < 0.001) and AIx (*p* = 0.04) with CVD in 138 RA patients [56]. Arterial stiffness was also found to be associated with cardiac involvement in RA, as cfPWV correlated with increasing filling pressure and impaired relaxation of the left ventricle [84]. Moreover, AIx (r = 0.334, *p* = 0.009) and cfPWV (r = 0.360, *p* = 0.005) were found to correlate with a higher myocardial performance index (a marker for cardiomyopathy) in a study of 40 RA patients evaluated for left ventricle dysfunction in the context of the disease [64].

It is well known that in RA, atherosclerosis is accelerated, as systemic inflammation in the context of the disease may promote atherogenic pathophysiologic mechanisms [85] However, Robustillo-Villarino et al. found no significant association between carotid IMT and either baPWV or AIx in 194 consecutive RA patients without established CVD [86]. This could be because arterial stiffness is the result of both endothelial dysfunction and degenerative vascular changes, meaning that atherosclerosis alone does not necessarily explain increased arterial stiffness. 

In summary, based on the available evidence, arterial stiffness seems to be significantly increased in RA patients and can be predicted using traditional CV risk factors. This result seems plausible, since RA patients are at high CV risk. Physical exercise, selected immunosuppressants, and some antihypertensive medications may have a positive effect on arterial stiffness and subsequently on arterial health status. However, data on the associations of arterial stiffness with markers of RA disease activity and chronicity are heterogeneous and should be examined further.

#### 4.2.2. Psoriatic Arthritis

PsA is another inflammatory musculoskeletal disease with a prevalence of 2–4% in Western adults [87]. Approximately 20–30% of patients with skin psoriasis develop PsA [87].

Patients with PsA often suffer from a number of comorbidities, such as bowel inflammation, skin/nail disease, depression, and osteoporosis [87]. Moreover, increased CV morbidity is described, including ischemic heart disease, congestive heart failure, and peripheral vascular disease [88]. CVD has also been described as the leading cause of death, accounting for about 38% of all deaths in PsA [89].

In PsA, aortic PWV is significantly increased compared to healthy controls, even after adjustment for the effect of confounding factors (in the study of Costa et al.: age, weight, height, heart rate, and central mean pressure; in the study of Shen et al.: age, gender, and body weight) [90,91]. Other markers of the decreased elasticity of vessels, such as left-ventricular (LV) stiffness and the aortic stiffness index, were also found to be abnormal in PsA patients [92]. However, the increase in aortic stiffness as represented by PWV or LV stiffness did not correlate with disease duration [90,91,92].

Costa et al. and Kristensen et al. found no significant association of aortic stiffness with disease activity as evaluated by DAS28, DAS66, or laboratory inflammation markers, such as ESR or CRP, in two cohorts of PsA patents (all *p* > 0.05) [90,93]. Conversely, Lo Gullo et al. found PWV to be associated with markers of disease activity (DAS28, BASDAI) in a small group of 35 PsA patients [94]. The case-control design and the small number of patients included make the extraction of valid statistical results difficult. However, in a prospective cohort study, Cheng et al. aimed to evaluate the effects of achieving minimal disease activity (MDA) on the progression of subclinical atherosclerosis and arterial stiffness in patients with PsA [95]. Interestingly, sustained MDA (defined as MDA at every visit between month 12 and month 24) was associated with the prevention of carotid atherosclerosis (odds ratio 0.273 (95% confidence interval 0.088–0.846), *p* = 0.024) and arterial stiffness progression in 101 consecutive patients with PsA [95].

In the study conducted by Shen et al., among patients with PsA, subgroups with high cfPWV values also showed a higher prevalence of traditional CV risk factors, such as systolic blood pressure, a higher Framingham score, diabetes, and hypertension (*n* = 34, all *p* < 0.05) [91]. This finding indicates that traditional CV risk factors should be also considered when treating patients with PsA to improve CV outcomes. Aside from aortic stiffness, patients with PsA also appear to exhibit a faster progression of subclinical atherosclerosis, as carotid intima-media thickness (cIMT) seems to be significantly higher in this group [96,97,98,99]. Kimhi et al. also found a higher cIMT to be associated with disease duration, as well as with disease-related parameters, such as spine involvement and higher fibrinogen, in a relatively large case control study involving 74 PsA patients [96].

Overall, data on arterial stiffness are limited in PsA, as studies on the topic are significantly fewer in comparison to RA. Moreover, most of the examined cohorts involved low numbers of patients (from *n* = 20 to *n* = 73 in 4 case control studies). Nevertheless, arterial stiffness also seems to be higher in PsA patients than in the general population, and both CV risk factors and the cumulative disease burden have been shown to predict this surrogate marker. It is hoped that new studies on the topic with more patients and standardized study designs will provide clearer insights in the future.

#### 4.2.3. Systemic Lupus Erythematosus

Systemic lupus erythematosus (SLE) is an autoimmune inflammatory disease, which often presents with multiorgan involvement [9]. Renal and neuropsychiatric manifestations are some of the most important clinical characteristics of the disease [100], while further common manifestations include skin lesions, photo-sensibility, vasculitis, and non-erosive polyarthritis [9]. SLE is associated with higher mortality compared to the general population, especially among young adults, with one of the main causes of death being CVD (next to renal disease and infections) [101].

A large body of evidence indicates arterial stiffness to be significantly higher in patients with SLE compared to healthy controls: patients with SLE have shown significantly higher values in PSEM [47,102,103], the stiffness index [104], AIx [104,105,106], and PWV (brachial–ankle PWV [65,107,108] carotid–femoral PWV [104,109,110,111]), as well as lower aortic and carotid distensibility [103,104]. On the other hand, some studies did not show increased arterial stiffness when comparing SLE patients to healthy controls, even after adjusting for confounders (in Stortz et al.: BMI, heart rate, hypertension, and lipids; in Tziomalos et al.: waist circumference, waist/hip ratio, alcohol consumption, triglycerides, fibrinogen, and GFR) [33,112,113]. 

The most important patient characteristics associated with increased arterial stiffness in SLE, as assessed by PWV, include age [33,65,108,109,111,112,114,115] and blood pressure [33,65,109,111,112,114,115]. PWV has also been shown to be associated with impaired kidney function, a lower estimated glomerular filtration rate (eGFR) [33,109], and lower creatinine clearance [114]. Moreover, our research group found an independent statistically significant inverse association between eGFR and cfPWV in SLE patients (β = −0.20; *p* = 0.033). This result is important, given that the examined population had a widely normally ranged eGFR [33]. Additional parameters associated with PWV in SLE are the disease duration [65,104], SLE activity (as assessed by SLEDAI) [108], inflammatory activity assessed by CRP [108,116], ESR [108], and IL-6 [116]. Moreover, different CV risk stratification scores, such as the Framingham risk score [65] (PWV: r = 0.61, *p* < 0.001), as well as resting heart rate (95% CI: 0.08–1.14; *p* = 0.024) [117], have been found to correlate with markers of arterial stiffness.

AIx has also been shown to correlate positively with age [105], blood pressure [104,105], inflammation markers (CRP, IL-6) [116], and the traditional CV risk assessment tool SCORE [105] in SLE patients. Moreover, AIx was predicted by age, immunoglobulin M-ß2-cardiolipin antibodies (IgM-ß2-GLP I), and a low number of small HDL particles [105].

Interestingly, arterial stiffness in the context of SLE seems to also be associated with more prominent atherogenesis. cfPWV correlated with the internal carotid wall thickness (*n* = 41; *p* = 0.0017) [109] and total cholesterol (*n* = 30; *p* < 0.001) [65] in SLE cohorts, and Parra et al. have also described an association between apolipoprotein B (ApoB) and AIx in 69 female SLE subjects [105]. Furthermore, ApoB has been shown to be a predictor of baPWV, next to age and blood pressure [114]. This finding is in line with reported higher cIMT values [47,104,105], correlating with QRISK3 [113] and with disease duration [104], as well as with the presence of atherosclerotic plaques [47].

While arterial stiffness is closely linked to age and seems to be more pronounced in patients with SLE, relative increases in arterial stiffness, assessed by PSEM, showed no difference between 76 SLE patients and 26 age/sex-matched healthy controls in a study conducted by Roldan et al. [47].

These tendencies for increased arterial stiffness are noteworthy, as stiffer arteries may be associated with impaired left ventricular (LV) function [106]. Higher levels of arterial stiffness have been shown to be associated with the increased concentric remodeling of the left ventricle [106], as well as with a higher LV mass and left atrium volume. Moreover, a dysfunction of the left heart was found to be independently associated with MAP, complement C3, and SLE duration (*n* = 76; all *p* < 0.04) [103]. 

Interestingly, endothelial dysfunction seems to play a role in the development of arterial stiffness, as patients with SLE display lower flow-mediated dilation (FMD) and higher levels of vascular cell adhesion molecule-1 (VCAM-1) compared with healthy controls [104,118]. In turn, VCAM-1 correlated with disease activity (as assessed by SLEDAI; rho = 0.246), ESR (rho = 0.263), and the anti-coagulation factor thrombomodulin (rho = 0.246), as well as with the pro-coagulatory tissue factor III (rho = 0.323), in a study of 127 SLE patients [118].

Interestingly, findings on the relationship between cardiorespiratory fitness and arterial stiffness in patients with SLE vary depending on the examined cohort and/or the chosen assessment marker of arterial stiffness [119,120,121]. For instance, cardiorespiratory fitness measured via the Siconolfi step test and the 6-minute walk test was inversely associated with arterial stiffness measured via PWV in a cohort of 49 female SLE patients (both *p* < 0.005) [119]. Similarly, in a further case control examination of 41 SLE patients, subjects who exercised frequently showed no difference in the aortic AIx compared to controls of the same age, gender, and anthropomorphic measurements (*p* = 0.51) [120]. On the other hand, SLE patients who did not exercise regularly had statistically significantly higher AIx values compared to the controls (24.7 ± 3.9% vs. 8.2 ± 1.8%) [120]. However, Soriano-Maldonado et al. did not find cardiorespiratory exercise to influence arterial stiffness directly over 12 weeks [121]. Nonetheless, regular exercise may still yield some benefits, as metabolic syndrome is not only significantly associated with SLE [111] but also with a significantly higher cfPWV [122,123]. In addition, metabolic syndrome is associated with higher levels of homocysteine, CRP, uric acid, and possibly cumulative disease damage assessed by SLICC/ACR [123].

Another important aspect is the effect of disease-modifying antirheumatic drugs on the arterial stiffness of patients with SLE. In the study conducted by Du et al., lowering disease activity (SLEDAI) in 51 SLE patients was associated with an overall improved CV risk, as evidenced by a reduction in PWV and an increase in HDL over a follow-up period of 1 year [124]. Furthermore, the treatment of SLE patients with antimalarial drugs was linked to significantly lower AIx values and a more favorable lipid profile (higher HDL) [105].

While the cardioprotective properties of lipid-lowering agents (statins) are well evidenced for the general population, the effects of this medication on arterial stiffness in patients with SLE have yet to be fully elucidated. Most importantly, longitudinal data on this topic are scarce. One of the few follow-up studies performed in an SLE cohort showed a statistically significant decrease in aortic stiffness (cfPWV) in the subgroup of patients with abnormal baseline cfPWV values after an 8-week course of therapy with atorvastatin at a dosage of 20 mg (8.43 ± 1.45 m/s vs. 7.42 ± 1.06 m/s; *p* = 0.002) [115]. On the contrary, Parra et al. found statistically significantly higher AIx values in SLE patients treated with statins [105]. However, the latter study had a cross-sectional design lacking follow-up examinations.

Another topic of frequent scientific discussions is the role of vitamin D (VitD) in SLE disease activity and arterial stiffening. There are reports in the literature that high disease activity (SLEDAI) may be associated with low VitD and, in turn, with a higher PWV and BMI [125]. Moreover, Sabio et al. found that patients from the lowest quartile of the 25-hydroxyvitamin D-range in a cohort of 106 nondiabetic SLE female patients had higher PWV values compared to those from the upper quartile [111]. However, there was no statistically significant association between 25-hydroxyvitamin D levels and PWV values [111]. On the other hand, Mellor-Pita et al. found that SLE patients with high levels of arterial stiffness also had higher levels of serum VitD, in most cases due to VitD/calcium supplementation [126]. As the authors stated, the results of these studies should be controlled in longitudinal, prospective explorations.

To summarize the SLE data presented here, the majority of these studies indicated increased arterial stiffness, even if statistical adjustments for the effects of confounding factors (including traditional CV risk factors) were not performed on every occasion. Interestingly, lowering disease activity can prove important in reducing arterial stiffness and thus the CV risk in SLE patients. One should keep in mind, however, that the thorough assessment and successful treatment of traditional CV risk factors are also essential steps towards improving CV and CVB prognosis.

#### 4.2.4. Systemic Sclerosis

SSc is a connective tissue disease with characteristic skin and organ fibrosis, as well as an obliterating vascular pathology [127]. It can be categorized into a limited cutaneous form (lcSSc) and a systemic diffuse one (dcSSc), the former being more common [127]. Typical symptoms and clinical signs are Raynaud’s phenomenon, telangiectasia, arthralgia, interstitial lung disease (80% of patients), and pulmonary arterial hypertension, as well as CV manifestations and cutaneous scleroderma [127]. The CV risk is significantly increased in these patients, especially due to the often unrecognized cardiac involvement [127].

In SSc, data on arterial stiffness are somewhat contradictory: some of the performed studies reported increased arterial stiffness as assessed by PWV [128,129,130,131], AIx [129,131,132], AoSI, or aortic strain and distensibility [133]. However, some more recent studies reported no statistically significant differences in arterial stiffness (PWV [132,134,135,136,137,138], AIx [136]) between SSc patients and controls. The heterogeneity of the studies’ methodologies, the low number of included patients, and likely systemic bias seem to be the causes of these contradictory results.

However, arterial stiffness has been found to be associated with various SSc disease- and patient-associated parameters. In addition to age [128,131,132,135,136], PWV has been shown to be associated with a lack of anti-Scl70 antibodies (*p* = 0.001) [132], increased blood pressure [131,132], treatment with ACE inhibitors (*p* = 0.034) [132], and homocysteine levels [138]. Disease duration has also been found to relate to higher PWV and AIx values [128,132,136]. PWV has been shown to be associated with anti-centromere antibodies [129,133] and with AIx among SSc patients [132]. Similarly, AIx was also found to be associated with anticentromere antibodies’ positivity, treatment with calcium canal blockers, blood pressure values [132], and a severe damage pattern in nailfold video-capillaroscopy (NVC) (all *p* < 0.05) [136]. Furthermore, AIx has been shown to be higher in active (34.1 ± 11.5%) and/or late (33.4 ± 8.8%) rather than early (20.5 ± 11.4%; *p* = 0.02 and *p* = 0.05, respectively) NVC patterns [135] and also to be associated with a lower density of capillaries per mm² (r = −0.34, *p* = 0.047) and a higher capillaroscopic skin ulcer risk index (CSURI) (r = 0.35, *p* = 0.044) [135]. However, Jung et al. did not find any statistical difference in arterial stiffness when comparing patients with different NVC patterns in a small cohort of 39 patients with SSc [131].

Notably, another marker of arterial stiffness (AoSI) did not correlate with SSc disease severity as measured by the Medsger score, but it did show associations with echocardiographic markers of impaired left ventricular diastolic function [133].

Regarding the effect of acute inflammatory activity on arterial stiffness, one SSc study reported that cfPWV could be predicted by CRP [134], whereas two further studies found no relationships between PWV and inflammation markers [133,135]. Aortic stiffness in SSc was also found to be predicted by age and blood pressure [134], as was the case in most healthy examined populations. SSc seems to be associated with lower heart rates, which is also a known influencing factor of PWV [129,133].

Patients with lcSSc exhibited higher values of AIx than patients with dsSSc, as shown by Bartoloni et al. (36 ± 8% vs. 24 ± 14%; *p* = 0.022) [134]. However, according to Sunbul et al., patients with lsSSc also tend to be significantly older [129]. Avenatti et al. did not find a statistical difference in cfPWV between subgroups of patients with the two distinct disease forms [136].

Examining markers of coronary vascular damage (coronary flow retention; CFR) and endothelial dysfunction (asymmetric dimethylarginine, ADMA), Turiel et al. found CFR to be lower and ADMA to be increased in patients with SSc [130]. These findings could indicate vascular damage of the coronary arteries, as well as generalized endothelial dysfunction in the included SSc cohort [130]. This finding is also in line with higher homocysteine levels as a marker of CV risk in patients with SSc [136].

Regarding the effects of medications on arterial stiffness in SSc, the data are inadequate. In a very small study (*n* = 5), Maslyanskiy et al. reported a significant decrease in both PWV and AIx after 6 months of treatment with rituximab [139]. In another small study (*n* = 9) by van Roon et al., treatment with Bosentan was also found to significantly decrease the forearm (brachial–radial) PWV in patients with SSc after periods of 3 and 12 months. However, cfPWV did not differ between the Bosentan group and the normal care group in the same study [137]. The small number of patients precludes the generalization of these results.

In summary, angiopathy is a well-established comorbidity of SSc, and arterial stiffness could prove to be a promising biomarker of vascular manifestations since associations with NVC and several disease-associated parameters have been shown. However, more thorough research including homogeneous study designs and more included subjects is needed.

## 5. Critical Appraisal of the Potential Utility of Arterial Stiffness Assessments

The data presented here indicate the probable diagnostic value of arterial stiffness as a surrogate CVD and atherosclerosis assessment marker in patients with the examined arthritides and connective tissue diseases. Large prospective studies examining its diagnostic accuracy in the CV risk assessment of rheumatic patients are lacking. However, arterial stiffness provides information on patients’ vascular health status in a non-invasive, radiation-free, and non-complicated manner. Moreover, it can reflect the long-term effects not only of well-established risk factors but also of inflammation-associated chronic vascular damage. However, most importantly, asymptomatic patients without past CV or CVB events could also be screened using this method in order to identify subclinical CV disease and allow for the early initiation of treatment.

## 6. Discussion

In rheumatic diseases, CVD is recognized as one of the leading causes of premature morbidity and mortality [140]. Even though a high CV risk is well established in patients with systemic autoimmune diseases, validated studies of CV assessment methods are relatively scarce. Recent ESC guidelines (2021) recommend considering CV risk assessment in all patients with systemic inflammatory conditions [141]. Moreover, it is suggested that such patients should be treated with interventions similar to those used for high-risk subjects from the general population (next to anti-inflammatory agents), since these therapeutic strategies also seem to reduce the risk of CVD in rheumatic populations [141]. Traditional CVD prediction scores, such as the Framingham score, PROCAM Score, and SCORE, have been thoroughly examined and validated in the general population [15,16,17]. However, none of these tools were specially developed for patients with systemic inflammatory diseases and thus do not take into account the ameliorating effects of inflammatory activity on the CV system. Moreover, the effects of rheumatic-disease-associated factors, such as glucocorticoid and immunosuppressive treatments, abnormal lipoprotein functions, endothelial dysfunction, increased vasospasm, and accelerated atherosclerosis, cannot be assessed by these scores. Therefore, using just these tools can lead to the misclassification (and often to the underestimation) of the CVD risk in patients with rheumatic diseases [34]. For these reasons, newer scores, such as QRISK3, account for the presence of RA or SLE as an additional risk parameter, in addition to well-established traditional CV risk factors. The combined use of QRISK3 and SCORE allowed for the identification of most RA patients at high risk of carotid plaques in the cohort of Corrales et al. [142]. Moreover, QRISK3 has demonstrated better performance in predicting the risk of CVD in SLE patients than traditional CV risk scores, such as the Framingham risk score [143]. However, QRISK3 is not validated for use in further rheumatic diseases, limiting its use to those two rheumatic conditions.

This review study aimed to present some of the research that examines the diagnostic value of arterial stiffness as a surrogate marker of CVD in patients with rheumatic diseases. Here, we focused on two common arthritides, RA and PsA, and two connective tissue diseases prone to multiorgan involvement and, in particular, CV involvement, i.e., SLE and SSc. Through a study of the literature, it became clear that the existing data are characterized by high heterogeneity and that more studies with larger cohorts and longitudinal designs are needed in order to reach clear conclusions regarding the diagnostic value of the different surrogate markers of arterial stiffness and CV risk. In particular, follow-up examinations and an evaluation of their associations with future CV endpoints would shed light on the true diagnostic value of arterial stiffness in patients with rheumatic diseases. Such a study is currently being performed in our clinic, aiming to determine the occurrence of CVD in rheumatic patients who have undergone cfPWV measurements in the past eight years. Moreover, connective tissue diseases, such as myositis, Sjögren’s syndrome, mixed connective tissue disease, and undifferentiated connective tissue disease, as well as vasculitides, should be more thoroughly examined in the future. A further limitation of using arterial stiffness as a surrogate marker is the fact that it can be confounded by different traditional CV risk factors (i.e., arterial pressure, age, heart rate) and disease characteristics. For this reason, carefully designed studies with matched case controls and/or adjusted statistical analyses are warranted. 

To conclude, markers of arterial stiffness, such as the pulse wave velocity of the aortic vasculature and AIx, have been thoroughly examined in the general population and also in rheumatic populations (mainly in the context of cross-sectional studies). Despite the aforementioned limitations, arterial stiffness provides additional information regarding the elastic properties of the examined arteries. Thus, it can be used as an additional (surrogate) marker of arterial status and CVD, in addition to well-established traditional CV risk factors and scores. Alternative markers of arterial stiffness also seem promising; however, further research is needed to determine whether these tools can also be integrated into screening algorithms. Moreover, well-designed longitudinal studies examining patients with systemic inflammatory conditions are needed. 

## Figures and Tables

**Table 1:** Overview of different measurement methods of arterial stiffness.

Marker of Arterial Stiffness	Obtained by
Pulse wave velocity	Measuring the pulse time delay between two different areas of the arterial tree
Augmentation index (or augmentation index 75)	Pulse wave analysis with standardized 75 bpm AIx = augmentation pressure/pulse pressure × 100
Pressure–strain elastic modulus	[k (SBP − DBP)/[(SD – DD)/DD], where k = 133.3 is the conversion factor from mmHg to Nm^−2^ (Pascal Units)
Aortic distensibility	(cm^2^/dyn) = 2 × (SD − DD)/[(SBP − DBP) × DD]
Aortic stiffness index	AoSI = ln (SBP/DBP)/(AoS − AoD)/AoD
Cardio-ankle vascular index	CAVI = *a*[(2*ρ*/SBP − DBP) × ln(SBP/DBP) × PWV*^2^*] + *b*

PWV: pulse wave velocity; AIx: augmentation index; bpm: heart rate in beats per minute; PSEM: pressure–strain elastic modulus; SBP: systolic blood pressure; DBP: diastolic blood pressure; SD: end-systolic diameter; DD: end-diastolic diameter; AoSI: aortic stiffness index; AoS: aortic systolic diameter; AoD: aortic diastolic diameter; CAVI: cardio-ankle vascular index; *ρ:* blood density; *a* and *b*: constants.

**Table 2:** Longitudinal studies examining the predictive value of arterial stiffness in patients with rheumatic diseases.

Reference	Rheumatic Disease	Marker(s) of Arterial Stiffness	Study Design	Study Population	Follow-Up	Statistical Analysis	Result
Cioffi et al., 2016 [57]	RA	AoSI	Prospective case control study	*n* = 226 RA patients and *n* = 226 non-RA patients, matched for CV risk factors	Median: 12 months	Multivariate Cox regression analysis for death or all-cause hospitalization—AoSI: HR 2.85 (95% CI 1.03–7.85) ROC: clinical model for abnormally high AoSI: AUC 0.67 (95% CI 0.59–0.78); *p* < 0.001, sensitivity 68%, specificity 65%	Abnormal AoSI predicted independently death or all-cause hospitalization
Ikdahl et al., 2016 [56]	RA	Aortic (cf) PWV and AIx	Prospective case study (no controls)	*n* = 138 patients	Mean: 5.4 years	Crude Cox proportional hazards regression of possible predictors of CVD events: cfPWV: HR 1.85 (1.33–2.57); *p* < 0.001 AIx: HR 1.05 (0.98–1.13); *p* = 0.19	cfPWV (and not AIx) was predictive of CVD events
Rosato et al., 2014 [58]	SSc	Doppler: Mean values of 3 measurements of interlobar arteries of each kidney: PSV, EDV, RI, PI, S/D	Prospective case control study	*n* = 70 patients and *n* = 30 healthy controls	3, 6, 9 and 12 months after baseline examination	DU prediction: ROC: RI: 0.94, (95% CI 0.87–1); *p <* 0.0001), S/D: 0.92, (95% CI 0.85–0.99); *p <* 0.0001), PI: 0.88, (95% CI 0.80–0.96); *p <* 0.0001. PSV and EDV non-significant (*p* > 0.05) PPV-RI (cut-off 0.70): 90.6% (95% CI 81.6–99.4%) and NPV 92.8% (95% CI 80.5–98.4%), PPV-S/D (cut-off 3.25): 92.9% (95% CI 76.5–98.9%) and NPV-S/D 90.58% (95% CI 77.4–97.3%)	Only RI, S/D, and PI showed good accuracy in predicting new DU. No statistically significant performance was found for PSV and EDV
Constans et al., 2007 [59]	SSc	QKd 100-60	Multicenter prospective study	*n* = 99 patients with at least one follow-up (*n* = 83 included in the multivariate analysis)	1, 2, and 3 years after inclusion—mean follow-up duration: 1103 days (SD = 205 days)	Multivariate analysis evaluating predictors of disease progression: QKd 100-60 < 200 ms: OR 19.6, (95% CI 1.2–308.2); *p* = 0.03	QKd 100-60 predicted severe progression * of systemic sclerosis

RA: rheumatoid arthritis; AoSI: aortic stiffness index; HR: hazard ratio; ROC: receiver operating characteristics; AUC: area under the curve; CI: confidence interval; cfPWV: carotid–femoral pulse wave velocity; AIx: augmentation index; CVD: cardiovascular disease; SSc: systemic sclerosis; PSV: peak systolic velocity; EDV: end diastolic velocity; RI: resistive index; PI: pulsatile index; S/D: systolic/diastolic ratio; DU: digital ulcerations; PPV: positive predictive value; NPV: negative predictive value; QKd: interval reflecting the time between onset of the QRS wave on electrocardiogram (Q) and the detection of the last Korotkoff’s (K) sound, corresponding to diastole (d). * Progression of SSc: skin or visceral progression as defined by Constans et al. [59].

## Data Availability

No new data were generated for this study.
